# A Nomogram Model for Early Mortality Risk Stratification in Elderly Patients with Idiopathic Pulmonary Fibrosis: An Integrative Analysis of Serum Biomarkers and Pulmonary Function Parameters

**DOI:** 10.3390/jcm15135124

**Published:** 2026-07-01

**Authors:** Yingying Zhu, Zhangyan Ke, Tiantian Zhang, Siyu Sun, Xiaoyun Fan

**Affiliations:** 1 Department of Geriatric Respiratory and Critical Care Medicine, The First Affiliated Hospital of Anhui Medical University, Hefei 230022, China; yfy1361043@fy.ahmu.edu.cn (Y.Z.);; 2 Anhui Provincial Institute of Geriatrics, The First Affiliated Hospital of Anhui Medical University, Hefei 230022, China

**Keywords:** elderly, idiopathic pulmonary fibrosis, nomogram, early mortality, serum biomarkers

## Abstract

**Background:** Idiopathic pulmonary fibrosis (IPF) has a low incidence but high mortality. Simple prognostic tools for elderly IPF patients in primary care are lacking. This study aimed to develop an accessible nomogram for this population. **Methods:** A retrospective analysis was conducted on elderly IPF patients from the First Affiliated Hospital of Anhui Medical University (January 2016–February 2023). Early mortality was defined as death within 12 months from diagnosis. A nomogram was developed using predictors identified by univariate and multivariate analyses. To minimize overfitting, we limited the number of predictors to four based on the rule of at least 10 events per variable (39 events). Model performance was assessed using the C-index, calibration curves including the Hosmer–Lemeshow goodness-of-fit test, ROC analysis, and decision curve analysis (DCA). Internal validation was performed using bootstrap resampling (1000 iterations). For missing data, variables with >30% missingness were excluded; for variables with ≤30% missingness, multiple imputation was applied. Risk stratification was performed based on nomogram scores, and survival between groups was compared via Kaplan–Meier analysis. **Results:** Overall, 83 patients were included. Multivariate analysis identified age, monocyte count, globulin, and DLCO%pre as independent predictors of early mortality. The nomogram incorporating these factors achieved a C-index of 0.846 (optimism-corrected C-index: 0.812 after bootstrap). The AUCs for predicting 1-, 2-, and 3-year overall survival were 0.879, 0.896, and 0.854, respectively. The Hosmer–Lemeshow test showed good calibration (*p* = 0.42, 0.38, and 0.51 for 1, 2, and 3 years). Kaplan–Meier analysis confirmed significantly worse survival in the high-risk group (*p* < 0.0001). **Conclusions:** We developed an accurate and practical nomogram to predict prognosis in elderly IPF patients, offering a useful risk-assessment tool for primary care settings. However, external validation in independent multicenter cohorts is required before clinical implementation. At its current stage, the model should be regarded as hypothesis-generating.

## 1. Introduction

Idiopathic pulmonary fibrosis (IPF) is the most common type of idiopathic interstitial pneumonia, with a global prevalence ranging from 0.33 to 4.51 per 10,000 individuals [1]. Due to the lack of clinically established effective treatments, its prognosis is poorer than other types of idiopathic interstitial pneumonia. Population aging is a reported risk factor for IPF, and its incidence gradually increases with age [2]. In China, the prevalence of IPF is approximately 7 per 100,000 in the 18–34 age group, soaring to 227.2 per 100,000 among those aged over 75 years [3], posing a significant public health challenge for the elderly population and society. The etiology of IPF remains unclear but is thought to involve genetic predisposition and environmental factors. It is essentially a chronic, diffuse inflammatory disease of the lower airways, leading to alveolar septal thickening and pulmonary fibrosis. The typical natural history of IPF involves slow disease progression, ultimately resulting in respiratory failure and death, with a median survival of 3 to 5 years [4] and a 5-year survival rate of approximately 20% to 40% [5,6,7], which is lower than that of most malignancies. Although antifibrotic drugs such as nintedanib and pirfenidone can slow disease progression, they cannot reverse established fibrosis or definitively improve long-term prognosis [8,9]. Two recent bibliometric analyses have summarized the evolving landscape of antifibrotic research in interstitial lung diseases [10,11]. Pirfenidone and nintedanib remain the first-line antifibrotic drugs for ILD. They slow disease progression and reduce FVC decline, yet cannot reverse pulmonary fibrosis or improve long-term prognosis. Both agents cause distinct adverse reactions, mainly gastrointestinal symptoms and skin lesions. At present, novel effective therapies are scarce, and studies focus on optimizing medication regimens and exploring curative strategies to halt ILD progression. The clinical course of IPF patients exhibits significant individual heterogeneity [12]. Consequently, the accurate identification of elderly patients at high risk for early mortality is imperative for timely transplant referral, which is crucial for improving clinical outcomes [13]. However, in China, key medical resources are relatively concentrated, diagnostic capabilities in remote areas are limited, and the capacity for predicting early mortality risk in elderly IPF patients is insufficient in some regions, leading many patients to miss the opportunity for lung transplantation assessment.

Peripheral blood parameters are routinely tested in primary healthcare institutions and are relatively accessible. Numerous previous studies have highlighted the prognostic value of peripheral blood markers in IPF. A retrospective, multicenter cohort study by Scott et al. [14] suggested that monocyte count is a potential prognostic biomarker in IPF, with a high monocyte count (>0.95 × 10^9^/L) significantly associated with poor outcomes. Kreuter et al. conducted a more detailed stratification of monocyte count thresholds, after adjusting for demographics, physiology, comorbidities, and long-term immunosuppressant use, showing that IPF patients with monocyte counts >0.60 × 10^9^/L had a higher risk of disease progression, all-cause mortality, and hospitalization within one year [15]. Previous research also indicated that IPF patients with a white blood cell count ≥9 × 10^9^/L had a significantly shorter median transplant-free survival compared to those with a count <9 × 10^9^/L (32.4 months vs. 50.5 months). This association remained significant after adjusting for Gender–Age–Physiology (GAP) stage, steroids, and antifibrotic drugs, whether analyzed as a continuous or dichotomous variable [16]. Furthermore, the neutrophil-to-lymphocyte ratio and lymphocyte-to-monocyte ratio have also been suggested to hold prognostic value in IPF [17,18]. These studies collectively demonstrate the important prognostic value of peripheral blood markers in IPF.

In addition to peripheral blood markers, pulmonary function tests (PFTs) are widely used in IPF patients. Nishiyama et al. observed initial PFTs in 114 IPF patients and found that besides forced vital capacity (FVC), the baseline ratio of forced expiratory volume in 1 s to FVC (FEV1/FVC) could also serve as a prognostic indicator for overall survival. Using a cutoff of 83%, patients with FEV1/FVC > 83% had shorter survival and worse prognosis. The difference in survival curves was more pronounced using a 70% cutoff, showing an inverse correlation between survival and FEV1/FVC [19]. Novelli et al. found that IPF patients with an initial FVC % predicted >72.5% had a better prognosis than those below this threshold, with the latter having a 16.4-fold higher risk of death, while diffusing capacity for carbon monoxide (DLCO) did not show similar prognostic value [20].

The establishment of the GAP staging system has significantly enhanced the capacity for mortality risk prediction in patients with idiopathic pulmonary fibrosis (IPF). Nevertheless, subsequent efforts to refine its prognostic accuracy often incorporate esoteric variables, such as genetic markers and specific inflammatory cytokines, which are not routinely accessible in clinical practice. Moreover, prognostic models specifically tailored for the elderly IPF population are notably lacking. The rationale for including tumor markers (CEA, CYFRA 21-1, NSE) in this IPF cohort was exploratory; IPF and lung cancer share common risk factors including age and smoking, and epithelial injury in IPF may lead to elevated CYFRA 21-1 levels [21,22]. Consequently, leveraging readily available parameters—specifically, standard serum biomarkers and pulmonary function tests (PFTs)—this study aims to develop an accessible risk stratification model to facilitate the identification of high-risk elderly IPF patients in primary care settings, enabling earlier transplant evaluation.

## 2. Materials and Methods

### 2.1. Standard Protocol Approvals, and Patient Consents

This study was approved by the Hospital Ethics Committee in the First Affiliation of Anhui Medical University and was carried out in accordance with the Declaration of Helsinki. All subjects signed informed consent (Ethics Approval Number: 2024792).

### 2.2. Study Population

This study screened 115 IPF patients treated at the First Affiliated Hospital of Anhui Medical University between January 2016 and February 2023. All patients had imaging suggestive of Usual Interstitial Pneumonia (UIP) and were diagnosed with IPF according to the updated 2022 American Thoracic Society/European Respiratory Society guidelines [23,24]. Early mortality was defined a priori as death within 12 months from the date of diagnosis. Collected indicators included complete blood count, liver and kidney function, pulmonary function test reports (including ventilation and diffusion capacity) and Lung Cancer Tumor Markers within seven days of the first visit. Arterial blood gas results were also added to optimize the model’s predictive value. Follow-up was conducted every six months until patient death or the study endpoint (February 2024). The follow-up protocol included standardized questionnaires regarding vital status, hospitalization, and new diagnoses. Exclusion criteria included: concurrent malignancy with an expected survival of less than one month; severe hepatic or renal dysfunction; hematopoietic disorders; long-term use of immunosuppressants or corticosteroids; incomplete data; and loss to follow-up during the study. For missing data, variables with >30% missingness were excluded from analysis. For variables with ≤30% missingness, multiple imputation by chained equations (MICE) with 5 imputations was performed.

### 2.3. Data Collection

**Figure 1:**
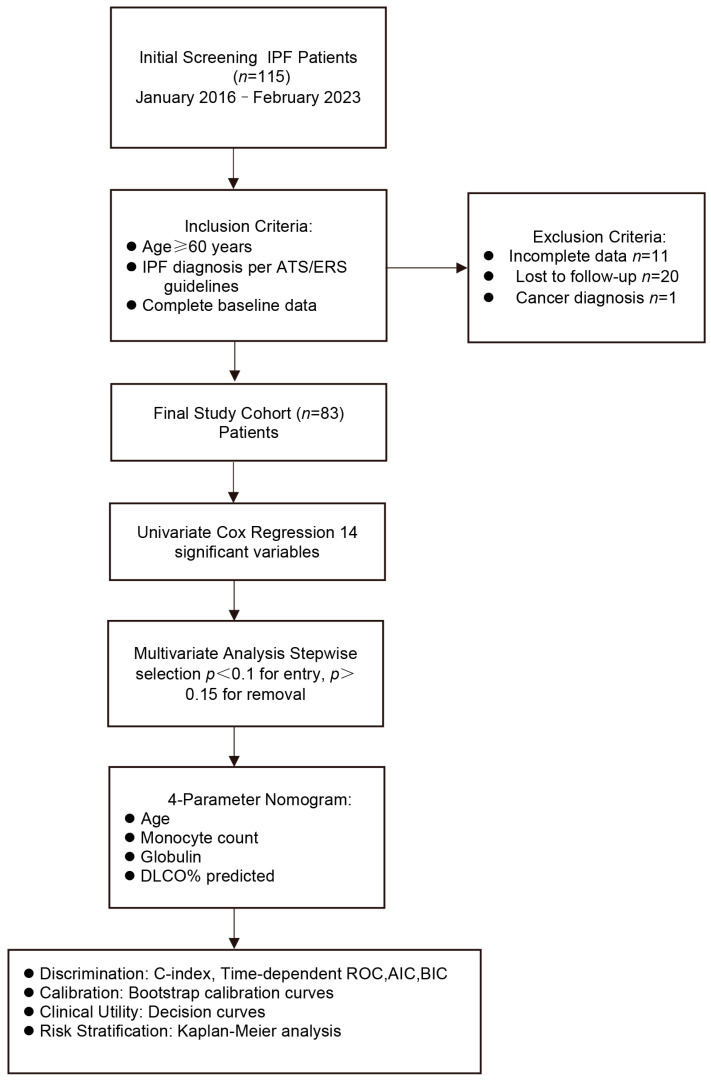
Patient Screening Flowchart.

We used serological test results and PFT indicators to predict early mortality risk in IPF patients. The following detailed information was collected from each enrolled patient’s medical records: sex, age, Body Mass Index (BMI), smoking history, White Blood Cell Count (WBC), Neutrophil Count (N), Monocyte Count (M), Eosinophil Count (E), Basophil Count (B), C-Reactive Protein (CRP), Total Protein (TP), Albumin (ALB), Globulin (GLB), PO_2_ (PaO_2_), Carcinoembryonic Antigen (CEA), Cytokeratin 19 Fragment (CYFRA 21-1), Neuron-Specific Enolase (NSE), Forced Vital Capacity (FVC), Forced Expiratory Volume in 1 s (FEV1), Maximum Voluntary Ventilation (MVV), Residual Volume to Total Lung Capacity Ratio (RV/TLC), and Diffusing Capacity for Carbon Monoxide Percent predicted (DLCO %pre). The primary endpoint was overall survival (OS), calculated from the date of diagnosis to the date of death or the last follow-up (February 2024). Initially, 115 IPF patients were enrolled. Eleven were excluded due to incomplete data. The remaining 104 patients were followed up via telephone and outpatient visits, among whom 20 were lost to follow-up and 1 was diagnosed with a new malignancy during follow-up. Thus, 83 patients were ultimately included in the analysis (Figure 1).

### 2.4. Model Construction and Statistical Analysis

All statistical analyses were performed using SPSS software (version 23.0) and R software (version 4.4.1 for Windows). Continuous variables are presented as median with interquartile range and were compared using independent samples *t*-tests. Categorical variables are presented as percentages and were compared using the χ^2^ test. Cox proportional hazards regression analysis was used to screen for prognostic factors for OS. Variables with a significance level of *p* < 0.05 in univariate analysis were included in a bidirectional stepwise Cox regression multivariate analysis (entry criteria *p* < 0.1, removal criteria *p* > 0.15). To ensure robustness given the sample size, we adhered to the rule of at least 10 events per predictor variable (39 deaths, maximum 4 predictors in the final model). Variance inflation factors (VIFs) were calculated to assess multicollinearity among selected variables; VIF > 5 was considered indicative of significant collinearity. Based on the multivariate analysis results, a nomogram was constructed to predict 1-year, 2-year, and 3-year OS. Internal validation was performed using bootstrap resampling with 1000 repetitions to calculate optimism-corrected C-index. Calibration was assessed using calibration curves and the Hosmer–Lemeshow goodness-of-fit test (*p* > 0.05 indicating good calibration). To test the goodness-of-fit of the nomogram, calibration curves were plotted using the bootstrap resampling method (1000 repetitions). A curve above the diagonal indicates that the predicted survival rate is lower than the actual survival rate, while a curve below the diagonal indicates overestimation. To assess the model’s discriminatory ability, time-dependent ROC curves were plotted, and the concordance index (C-index), Akaike Information Criterion (AIC), and Bayesian Information Criterion (BIC) were calculated. The C-index compares whether the risk order predicted by the model is consistent with the actual survival time order, ranging from 0.5 to 1, with values closer to 1 indicating better discrimination. No adjustment for multiple testing was performed due to the exploratory nature of the study; this is acknowledged as a limitation. AIC and BIC are comprehensive indicators measuring model fit and complexity; lower values indicate a better balance between fitting ability and parsimony. Additionally, decision curve analysis (DCA) was performed to evaluate the clinical utility of the nomogram. The Kaplan–Meier method and log-rank test were applied for calculating and comparing differences in OS between risk groups.

## 3. Results

### 3.1. Baseline Characteristics of the Study Population

Eighty-four IPF patients were followed up via telephone or medical record review. One patient was diagnosed with advanced lung cancer during follow-up and was excluded from the study cohort, resulting in 83 participants. Telephone follow-up revealed that 39 patients had died by the end of the follow-up period. Among the 115 initially screened patients, 11 were excluded due to incomplete data, 20 were lost to follow-up (19.2% attrition rate), and 1 was diagnosed with advanced lung cancer during follow-up. Statistically significant differences (*p* < 0.05) were observed between the survival and death groups in age, N, GLB, PaO_2_, CEA, CYFRA21-1, VCmax, FVC, FEV1, RV/TLC, and DLCO%pre. The basic characteristics of the enrolled IPF patients are presented in Table 1.

### 3.2. Univariate and Multivariate Analyses

Univariate analysis results showed that smoking history, age, WBC, M, GLB, PCO_2_, PO_2_, CEA, CYFRA21-1, VCmax, FVC, FEV1, RV/TLC, and DLCO%pre were statistically significant. Bidirectional stepwise Cox regression (entry *p* < 0.1, removal *p* > 0.15) was used to select variables from those significant in univariate analysis. The results identified age, M, GLB, CYFRA21-1, RV/TLC, and DLCO%pre as key variables. Following discussion with clinical specialists, several variables with weaker clinical relevance were excluded, and the final variables selected for model construction were age, M, GLB and DLCO%pre. VIFs for the four final variables were 1.24, 1.18, 1.43, and 1.02, respectively, indicating no significant multicollinearity. The results are shown in Table 2.

### 3.3. Nomogram Construction

**Figure 2:**
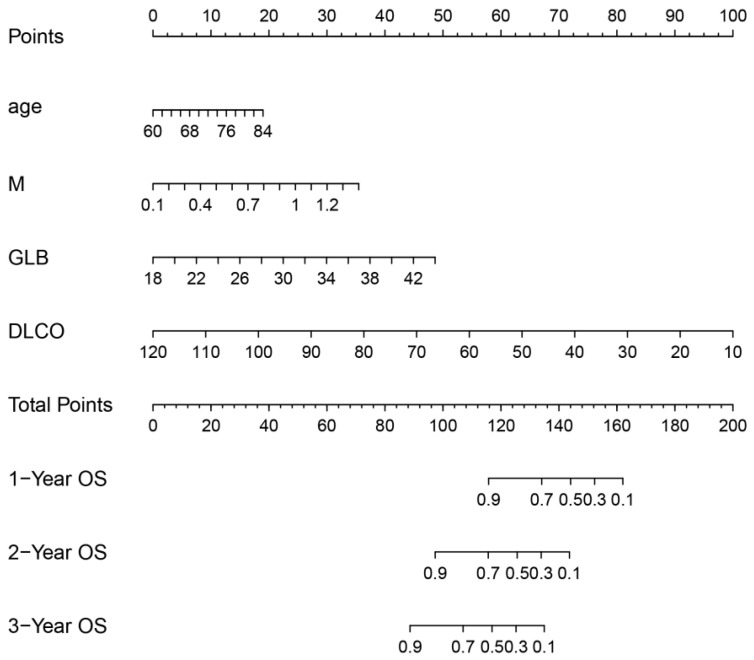
Nomogram Prediction Model for Survival in Elderly PF Patients. Note: M: Monocyte Count; GLB: Globulin; DLCO%pred: Diffusing Capacity for Carbon Monoxide percent predicted.

By integrating the prognostic markers age, M, GLB, and DLCO%pre, we constructed a nomogram for predicting 1-year, 2-year, and 3-year OS (Figure 2). In this nomogram, each prognostic indicator corresponds to a point on the “Points” axis, obtained by drawing a vertical line upward from the specific value of each indicator. To calculate the 1-year, 2-year, and 3-year OS probability for a specific patient, sum the points for all prognostic indicators to get the “Total Points,” then draw a line downward from this sum to the axes labeled “1-year OS,” “2-year OS,” and “3-year OS.” A higher total point score indicates a worse prognosis.

### 3.4. Model Performance

**Figure 3:**
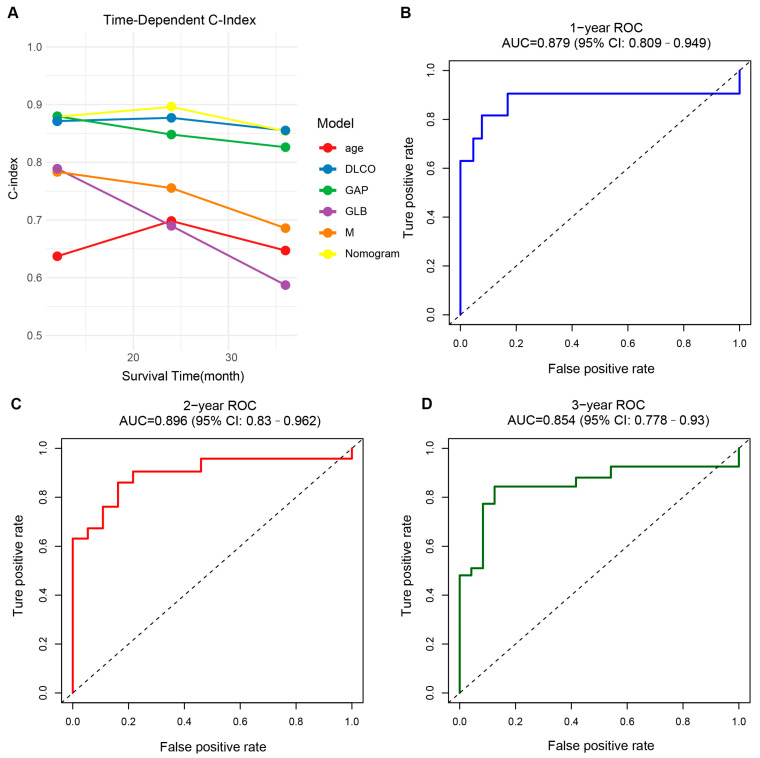
Concordance Index of the Nomogram and Receiver Operating Characteristic Curves. (**A**): Comparison of time-dependent concordance indices between the nomogram, GAP-model and individual indicators for predicting overall survival in IPF patients; (**B**): Area under the ROC curve for predicting 1-year overall survival, value 0.879; (**C**): Area under the ROC curve for predicting 2-year overall survival, value 0.896; (**D**): Area under the ROC curve for predicting 3-year overall survival, value 0.854. Note: C-index: Concordance Index; ROC: Receiver Operating Characteristic; AUC: Area Under the Curve; CI: Confidence Interval.

Time-dependent C-index analysis showed that compared to the GAP model and any single prognostic marker, the nomogram model demonstrated good prognostic accuracy and superiority in predicting patient 1-year, 2-year, and 3-year OS (Figure 3A). After bootstrap optimism correction, the C-index was 0.812. We also calculated the Akaike Information Criterion (AIC) and Bayesian Information Criterion (BIC) for all models, including the GAP model. The model comparison results are shown in Table 3: our constructed model achieved the lowest AIC (246.33) and BIC (252.88). To further evaluate the predictive accuracy of the nomogram model, we plotted time-dependent receiver operating characteristic (ROC) curves for predicting 1-year, 2-year, and 3-year survival in IPF patients. The results showed that the model exhibited excellent discriminatory ability at all time points. As shown in Figure 3B–D, the AUCs for the model predictions were: 0.879 (95% CI: 0.809–0.949), 0.896 (95% CI: 0.83–0.962), and 0.854 (95% CI: 0.778–0.93) for 1, 2, and 3 years, respectively. This suggests that the model has highly precise discriminatory ability for short-term (1- and 2-year) prognosis prediction, and its predictive performance remains at an excellent level even for longer-term (3-year) prediction.

### 3.5. Calibration and Clinical Utility

**Figure 4:**
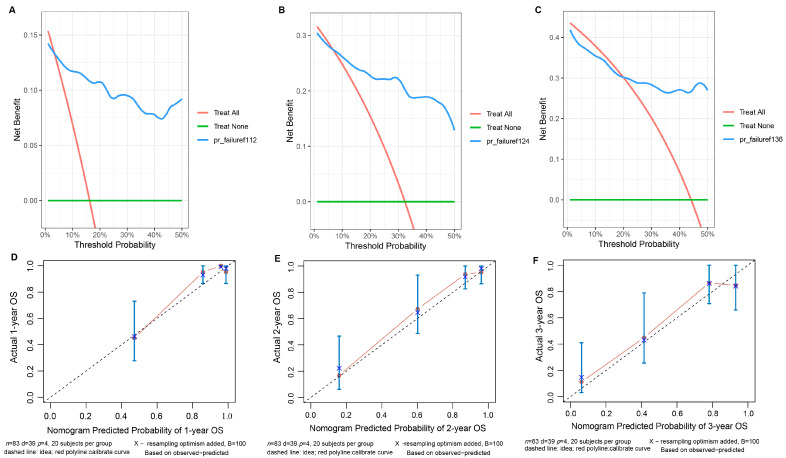
Calibration and Decision Curve Analysis of a Nomogram for Predicting 1-, 2-, and 3-Year Overall Survival. (**A**): Decision curve analysis results for 1-year overall survival; (**B**): Decision curve analysis results for 2-year overall survival; (**C**): Decision curve analysis results for 3-year overall survival; (**D**): Calibration curve for model prediction of 1-year overall survival; (**E**): Calibration curve for model prediction of 2-year overall survival; (**F**): Calibration curve for model prediction of 3-year overall survival.

The Hosmer–Lemeshow goodness-of-fit test showed no significant lack of fit for 1-year (*p* = 0.42), 2-year (*p* = 0.27), and 3-year (*p* = 0.51) predictions, indicating good calibration (Supplementary File S1). We assessed the net benefit and predictive ability of the nomogram using DCA and calibration curves. DCA for 1-year, 2-year, and 3-year OS (Figure 4A–C) indicated that across most of the reasonable probability threshold range, the nomogram provided a higher overall net benefit compared to single prognostic factors. Furthermore, calibration analysis visually presented the concordance between the nomogram-predicted risk and the actually observed risk through calibration plots. Figure 4D–F clearly demonstrates the good calibration performance of our nomogram in predicting 1-year, 2-year, and 3-year OS. Collectively, these results demonstrate that the validated nomogram provides robust predictive performance for survival prognosis.

### 3.6. Risk Stratification

**Figure 5:**
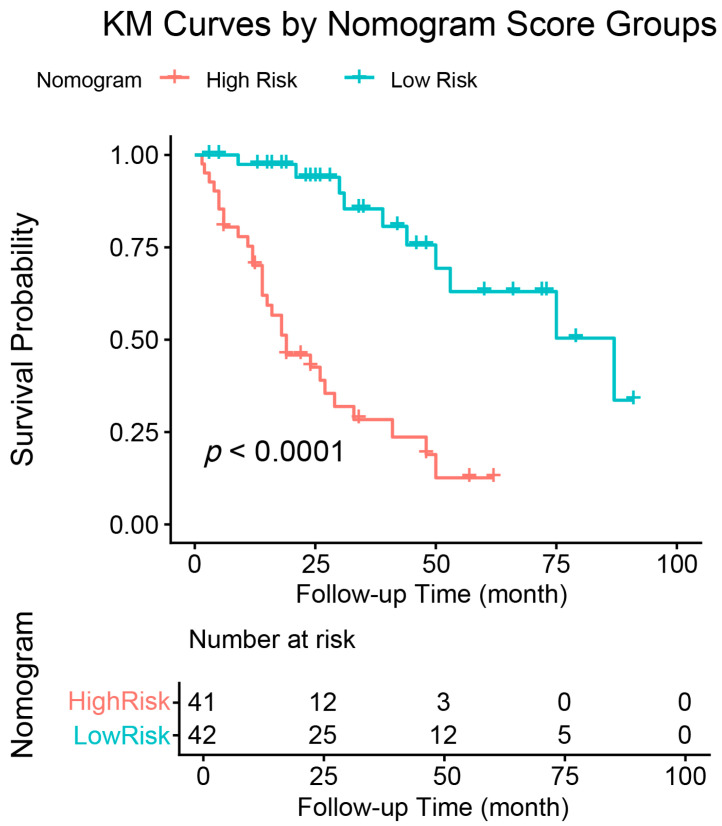
Kaplan–Meier Survival Curves for Nomogram-Based Risk Stratification (High-Risk vs. Low-Risk Groups).

To evaluate whether the nomogram could effectively distinguish elderly IPF patients with different risks based on OS, we calculated the total score for each patient. Patients were divided into low-risk and high-risk groups based on the median score. Kaplan–Meier analysis results showed that patients in the high-risk group had a significantly shorter OS compared to the low-risk group (*p* < 0.0001; Figure 5).

## 4. Discussion

IPF is a chronic progressive interstitial lung disease of unknown cause, characterized by repeated lung tissue injury and fibrosis formation, leading to progressive deterioration of lung function. It primarily affects middle-aged and elderly individuals. With the global aging trend, the number of elderly IPF patients is increasing annually. According to the “Report on the Illness Experience and Quality of Life of Patients with Idiopathic Pulmonary Fibrosis or Progressive Pulmonary Fibrosis in China (2024)” estimates, the annual number of new IPF cases in China is approximately 15,600 to 54,100 (based on East Asian data, annual incidence 1.2–4.16/100,000). Considering China’s large population base, aging process, and rising incidence, the disease burden imposed by these IPF patients on families and public health resources cannot be underestimated. Developing a simple and feasible early mortality risk prediction model holds significant clinical importance for clinicians in remote areas to refer high-risk patients for lung transplantation listing promptly. Previous studies often incorporated various indicators difficult to obtain in primary settings, such as single-cell sequencing results, transcriptome sequencing results, and pericardial fat measurements [25,26,27]. In this study, all parameters included in the model are common clinical indicators. The prediction model constructed from these four independent risk factors—age, M, GLB, and DLCO%pre—demonstrated higher predictive value than conventional prediction models. Compared to previous models, these parameters are simpler to obtain clinically and more operationally feasible.

Although nintedanib and pirfenidone are approved for IPF treatment and can delay fibrosis progression, neither antifibrotic drug can reverse the prognosis of IPF [8,9]. Lung transplantation, as the only method to improve the prognosis of IPF patients, has its optimal timing still undetermined. The GAP staging system, proposed by Ley et al. in 2012, identifies stage III patients as having high mortality and being strong candidates for immediate transplantation [28]. Furthermore, Collard et al. suggested in 2014 that survivors of an acute exacerbation of IPF (AE-IPF) should be considered for urgent transplantation [29]. Although clinically valuable, these studies encompass the general IPF population and lack specificity. Given that elderly patients exhibit distinct characteristics in disease physiology, aging status, and treatment responses, developing a prognostic model tailored to this specific subgroup could offer distinct advantages. This is the first model to incorporate monocyte count and globulin level for predicting mortality risk in IPF.

This is the first model to incorporate monocyte count and globulin level for predicting mortality risk in IPF. These variables enhance the model’s predictive power and offer novel clinical insights into disease progression from an immunological perspective. Monocytes, key mediators of innate immunity and a primary source of pulmonary macrophages, are continuously recruited to the lungs in IPF. There, they can differentiate into pro-fibrotic M2 macrophages, which directly drive fibroblast activation and proliferation through the secretion of potent mediators like TGF-β and PDGF, thereby exacerbating pulmonary fibrosis [30,31,32]. Consequently, an elevated peripheral monocyte count may reflect a more active, innate immunity-driven pro-fibrotic state, providing a biological basis for worse survival. Globulin, representing the sum of serum immunoglobulins, elevates during broad humoral immune activation and chronic dysregulation. Growing evidence implicates autoimmune processes in IPF pathogenesis, moving beyond the concept of a purely ‘non-inflammatory’ fibrotic disease [33]. This is supported by the detection of autoantibodies [34] and elevated IgA [35] in some patients. Our findings strengthen this view from a prognostic standpoint, suggesting that B-cell activation and antibody production are closely linked to aggressive disease. Thus, globulin level serves as a composite, quantifiable measure of the ‘immune dysregulation burden’. Incorporating these two indicators provides risk stratification complementary to traditional pulmonary function tests, reflecting distinct immunological pathways—innate (monocytes) and adaptive humoral (globulin) immunity.

Integration into clinical decision-making in resource-limited settings: In primary care settings where pulmonary function testing may be unavailable, simple thresholds for M (>0.6 × 10^9^/L, based on Kreuter et al. [15]) and GLB (>30 g/L, derived from the upper quartile in our cohort) can be used for initial risk triage. A total nomogram score >150 points (median split) defines the high-risk group. For such patients, we provisionally suggest referral for lung transplant evaluation within 3 months. These thresholds require prospective validation.

Regarding CYFRA 21-1, while multivariate analysis showed its prognostic value, we excluded it from the final model due to potential collinearity with DLCO%pre and concerns about age-related baseline shifts in elderly patients that may alter tumor marker interpretability. However, we agree that CYFRA 21-1 has biological plausibility as a marker of epithelial injury [22]. Future larger studies should evaluate a multi-biomarker panel including CYFRA 21-1, particularly in younger IPF patients or those with acute exacerbations.

Furthermore, multiple previous prediction model studies have mentioned age as a prognostic factor, consistent with our multivariate analysis results, indicating that age is a recognized element in assessing mortality risk in elderly IPF patients. Among pulmonary function test indicators, both RV/TLC and DLCO%pre are considered to have value in prognostic prediction for IPF patients. In the IPF-PRO study, a decision model including DLCO%pre, oxygen therapy use, and FVC % predicted helped predict survival beyond 5 years [36]. RV/TLC primarily reflects air trapping in the lungs. A study by Taehun Kim et al. suggested that an elevated RV/TLC is negatively correlated with pulmonary function and associated with adverse clinical outcomes in IPF, although it could not serve as an independent prognostic predictor [37]. Our multivariate analysis also highlighted the prognostic value of RV/TLC and DLCO%pre. However, considering the physiological changes related to aging, such as decreased lung elastic recoil and premature small airway closure, DLCO%pre was ultimately chosen for the final model.

Multivariate analysis also indicated the predictive value of CYFRA 21-1, a conventional tumor marker used in lung cancer screening. Oldham et al. performed proteomic profiling in IPF patients and identified keratin 19—the protein detected by the CYFRA 21-1 assay—as a biomarker associated with an increased risk of death or lung transplantation [21]. Similarly, a study by Ba et al. suggested that CYFRA 21-1 could predict prognosis in patients with acute exacerbation of interstitial lung disease (AE-ILD), regardless of the underlying subtype [38]. However, during model development, we considered that this antigen primarily reflects epithelial cell injury and abnormal hyperplasia. Given that persistent alveolar epithelial injury is a central pathogenic event in IPF [22], elevated CYFRA 21-1 levels are likely highly collinear with declining pulmonary function parameters, such as DLCO% predicted. Thus, it may be more a ‘consequence’ of disease activity than an independent ‘cause’. Furthermore, since our study specifically focused on elderly IPF patients, in whom non-specific chronic conditions and age-related physiological changes can alter tumor marker baselines, we questioned its discriminative power and clinical interpretability in this population. To develop a more specific and reliable prognostic model for elderly IPF patients, we therefore opted to exclude it.

Limitations: This study has several limitations. First, the sample size (*n* = 83) is relatively small, which may lead to overfitting. We attempted to mitigate this by limiting predictors to four (following the 10 events per variable rule), using bootstrap internal validation (optimism-corrected C-index: 0.812), and explicitly acknowledging the hypothesis-generating nature of the model. Second, this is a single-center study, limiting generalizability. We have initiated plans for external validation in two additional centers in Anhui Province; results will be reported separately. Third, the retrospective design introduces potential biases: (a) selection bias due to exclusion of incomplete records; (b) attrition bias (19.2% loss to follow-up); and (c) lack of treatment standardization (antifibrotic use was recorded but not controlled for). Fourth, DLCO%pre may not be available in all primary care settings, limiting the model’s applicability in the most resource-limited environments. A simplified model using only age, M, and GLB (excluding DLCO%pre) is provided as a sensitivity analysis in Supplementary Table S1. Fifth, no adjustment for multiple testing was performed due to the exploratory nature of the study. Finally, external validation in independent, multicenter cohorts is required before clinical adoption. At present, this model should be regarded as hypothesis-generating.

## 5. Conclusions

This study developed a predictive model by analyzing easily accessible and prognostically valuable indicators in elderly IPF patients. The model demonstrates good internal validity but requires external validation before routine clinical use. It should currently be considered hypothesis-generating.

## Figures and Tables

**Table 1:** Baseline Characteristics of Enrolled Patients.

Variable	Survival Group (*n* = 44, %)	Dead Group (*n* = 39, %)	t/χ2	*p*-Value
gender, n (%)			0.893	0.345
male	31 (70%)	31 (79%)		
female	13 (30%)	8 (21%)		
smoke, n (%)			2.79	0.124
yes	19 (43%)	24 (62%)		
no	25 (57%)	15 (38%)		
age (year, x ± s)	68.57 ± 5.75	71.59 ± 6.34	−2.276	0.026
BMI (kg/m^2^, x ± s)	23.55 ± 3.67	23.13 ± 4.36	0.485	0.629
WBC (109/L, x ± s)	6.76 ± 2.20	7.74 ± 2.92	−1.736	0.086
N (109/L, x ± s)	4.19 ± 1.83	5.21 ± 2.54	−2.115	0.037
L (109/L, x ± s)	1.82 ± 0.55	1.78 ± 0.77	0.247	0.805
M (109/L, x ± s)	0.51 ± 0.22	0.57 ± 0.21	−1.280	0.204
E (109/L, x ± s)	0.24 ± 0.17	0.25 ± 0.22	−0.216	0.830
CRP (mg/L, x ± s)	8.39 ± 19.76	11.22 ± 16.73	−0.701	0.485
TP (g/L, x ± s)	65.11 ± 5.15	66.70 ± 6.21	−1.270	0.208
ALB (g/L, x ± s)	38.69 ± 3.89	37.15 ± 4.00	1.773	0.080
GLB (g/L, x ± s)	26.42 ± 3.57	29.54 ± 5.73	−2.938	0.005
PCO_2_ (mmHg, x ± s)	38.14 ± 5.59	39.00 ± 6.04	−0.675	0.501
PO_2_ (mmHg, x ± s)	90.48 ± 19.28	83.09 ± 12.12	2.060	0.043
CEA (ng/mL, x ± s)	3.78 ± 2.40	5.50 ± 4.66	−2.140	0.035
NSE (ng/mL, x ± s)	14.15 ± 5.22	14.68 ± 5.02	−0.474	0.636
CYFRA21-1 (ng/mL, x ± s)	4.21 ± 1.85	5.08 ± 1.98	−2.081	0.041
VCmax (L, x ± s)	2.17 ± 0.71	1.76 ± 0.61	2.835	0.006
FVC (L, x ± s)	2.09 ± 0.70	1.69 ± 0.60	2.790	0.007
FEV1 (L, x ± s)	1.82 ± 0.62	1.50 ± 0.46	2.622	0.010
PEF (L/s, x ± s)	4.50 ± 2.12	4.23 ± 2.06	0.581	0.563
MVV (L/min, x ± s)	64.57 ± 23.60	59.36 ± 22.18	1.033	0.305
RV/TLC (%, x ± s)	43.78 ± 10.40	54.11 ± 13.71	−3.802	<0.001
DLCO%pre (%, x ± s)	47.58 ± 14.74	32.26 ± 22.73	3.684	<0.001

Note: BMI: Body Mass Index; WBC: White Blood Cell Count; N: Neutrophil Count; M: Monocyte Count; E: Eosinophil Count; B: Basophil Count; CRP: C-Reactive Protein; TP: Total Protein; ALB: Albumin; GLB: Globulin; PaO_2_: PO_2_; CEA: Carcinoembryonic Antigen; CYFRA 21-1: Cytokeratin 19 Fragment; NSE: Neuron-Specific Enolase; FVC: Forced Vital Capacity; FEV1: Forced Expiratory Volume in 1 s; MVV: Maximum Voluntary Ventilation; RV/TLC: Residual Volume to Total Lung Capacity Ratio; DLCO %pre: Diffusing Capacity for Carbon Monoxide Percent predicted.

**Table 2:** Univariate and multivariate Cox proportional hazards regression analysis for OS.

Variable	Univariate Analysis		Multivariate Analysis	
	HR (95% CI)	*p*-Value	HR (95% CI)	*p*-Value
gender				
male	1			
female	0.586 (0.265–1.293)	0.186		
smoke				
yes	2.322 (1.182–4.563)	0.014		
no	1			
age	1.078 (1.023–1.136)	0.005	1.084 (1.016–1.156)	0.014
BMI	0.967 (0.890–1.052)	0.436		
WBC	1.113 (1.010–1.226)	0.030		
N	1.104 (0.998–1.221)	0.054		
L	1.368 (0.757–2.474)	0.300		
M	5.783 (1.759–19.006)	0.004	10.541 (2.168–51.266)	0.004
E	3.663 (0.687–19.538)	0.129		
CRP	1.006 (0.995–1.018)	0.289		
TP	1.036 (0.979–1.097)	0.223		
ALB	0.945 (0.880–1.015)	0.118		
GLB	1.126 (1.052–1.206)	<0.001	1.114 (1.026–1.210)	0.01
PCO_2_	1.076 (1.010–1.147)	0.023		
PO_2_	0.954 (0.928–0.982)	0.001		
CEA	1.094 (1.016–1.177)	0.017		
NSE	1.032 (0.967–1.102)	0.342		
CYFRA21-1	1.259 (1.089–1.454)	0.002	1.321 (1.069–1.631)	0.01
VCmax	0.582 (0.376–0.900)	0.015	0.028 (0–3.924)	0.156
FVC	0.603 (0.392–0.928)	0.022	54.481 (0.379–7823.104)	0.115
FEV1	0.568 (0.342–0.943)	0.029		
PEF	0.934 (0.806–1.083)	0.365		
MVV	0.990 (0.976–1.004)	0.164		
RV/TLC	1.042 (1.020–1.064)	<0.001	1.052 (1.023–1.082)	<0.001
DLCO%pre	0.941 (0.918–0.965)	<0.001	0.962 (0.932–0.993)	0.015

Note: HR: Hazard Ratio; CI: Confidence Interval; BMI: Body Mass Index; WBC: White Blood Cell Count; N: Neutrophil Count; M: Monocyte Count; E: Eosinophil Count; B: Basophil Count; CRP: C-Reactive Protein; TP: Total Protein; ALB: Albumin; GLB: Globulin; PaO_2_: PO_2_; CEA: Carcinoembryonic Antigen; CYFRA 21-1: Cytokeratin 19 Fragment; NSE: Neuron-Specific Enolase; FVC: Forced Vital Capacity; FEV1: Forced Expiratory Volume in 1 s; MVV: Maximum Voluntary Ventilation; RV/TLC: Residual Volume to Total Lung Capacity Ratio; DLCO %pre: Diffusing Capacity for Carbon Monoxide Percent predicted.

**Table 3:** AIC, BIC, and C-index Values for the Nomogram and Comparison Models.

	C-Index	AIC	BIC
Nomogram	0.846	246.23	252.88
GAP	0.814	254.7	261.36
age	0.638	280.76	282.42
M	0.705	281.67	283.34
GLB	0.664	277.87	279.53
DLCO%pre	0.821	259.74	261.4

## Data Availability

The datasets generated and analyzed during the current study are available from the corresponding author on reasonable request.

## Supplementary Materials

The following supporting information can be downloaded at: https://www.mdpi.com/article/10.3390/jcm15135124/s1, Supplementary File S1: Hosmer–Lemeshow Goodness-of-Fit Test for Cox Model; Supplementary Table S1: Simplified Model Using Only Age, Monocyte Count, and Globulin (Excluding DLCO%pre).
