# Supplementary material for: A Nomogram Model for Early Mortality Risk Stratification in Elderly Patients with Idiopathic Pulmonary Fibrosis: An Integrative Analysis of Serum Biomarkers and Pulmonary Function Parameters
Source: J Clin Med. 2026 Jul 1;15(13):5124. doi: 10.3390/jcm15135124 (PMC13363289; doi:10.3390/jcm15135124)
Supplement: Supplementary file 1 [file jcm-15-05124-s001.zip › Supplementary Table S1-Simplified Model Using Only Age, Monocyte Count, and Globulin (Excluding DLCO%pre).pdf]

Supplementary Table S1

Supplementary Table S1. Simplified Model Using Only Age, Monocyte Count, and Globulin (Excluding DLCO%pre)

Model	C-index	AIC	BIC	1-year AUC (95% CI)	2-year AUC (95% CI)	3-year AUC (95% CI)
Full model (age + M + GLB + DLCO%pre)	0.846	246.23	252.88	0.879 (0.809-0.949)	0.896 (0.83-0.962)	0.854 (0.778-0.93)
**Simplified model (age + M + GLB)**	**0.801**	**254.67**	**260.15**	**0.841 (0.765-0.917)**	**0.858 (0.787-0.929)**	**0.819 (0.738-0.900)**

Note: The simplified model includes only age, monocyte count (M), and globulin (GLB), excluding DLCO%pre. This model may be more applicable in primary care settings where pulmonary function testing is unavailable. While its predictive performance is slightly lower than the full model, it still demonstrates acceptable discrimination (C-index = 0.801) and may serve as an alternative for initial risk triage in resource-limited environments.

Simplified Nomogram Scoring System (age + M + GLB only):

Variable	Points
Age (per year)	2 points per year above 60
Monocyte count (M) > 0.6 × 10^9/L	25 points
Globulin (GLB) > 30 g/L	20 points

Risk classification based on simplified model total score:

- Low risk: < 50 points
- Intermediate risk: 50-80 points
- High risk: > 80 points

These thresholds are provisional and require prospective validation.
